# Emerging Links between Cadmium Exposure and Insulin Resistance: Human, Animal, and Cell Study Data

**DOI:** 10.3390/toxics8030063

**Published:** 2020-08-27

**Authors:** Aleksandra Buha, Danijela Đukić-Ćosić, Marijana Ćurčić, Zorica Bulat, Biljana Antonijević, Jean-Marc Moulis, Marina Goumenou, David Wallace

**Affiliations:** 1 Department of Toxicology “Akademik Danilo Soldatović”, University of Belgrade-Faculty of Pharmacy, 11000 Belgrade, Serbia; danijela.djukic.cosic@pharmacy.bg.ac.rs (D.Đ.-Ć.); marijana.curcic@pharmacy.bg.ac.rs (M.Ć.); zorica.bulat@pharmacy.bg.ac.rs (Z.B.); biljana.antonijevic@pharmacy.bg.ac.rs (B.A.); 2 Alternative Energies and Atomic Energy Commission—Fundamental Research Division—Interdisciplinary Research Institute of Grenoble (CEA-IRIG), University of Grenoble Alpes, F-38000 Grenoble, France; jean-marc.moulis@cea.fr; 3 Laboratory of Fundamental and Applied Bioenergetics (LBFA), University of Grenoble Alpes, Inserm U1055, F-38000 Grenoble, France; 4 Centre of Toxicology and Forensic Sciences, Medicine School, University of Crete, 70013 Heraklion, Greece; marina.goumenou@gmail.com; 5 General Chemical State Laboratory of Greek Republic, 71202 Heraklion, Greece; 6 Department of Pharmacology & Toxicology, Oklahoma State University Center for Health Sciences, Tulsa, OK 74107, USA; david.wallace@okstate.edu

**Keywords:** cadmium, insulin, diabetes, hyperglycemia, hyperinsulinemia, lipogenic, β-cell toxicity

## Abstract

Recent research has helped clarify the role of cadmium (Cd) in various pathological states. We have demonstrated Cd involvement in pancreatic cancer, as well as the bioaccumulation of Cd in the pancreas. Bioaccumulation and increased toxicity suggest that Cd may also be involved in other pancreas-mediated diseases, like diabetes. Cd falls into the category of “hyperglycemic” metals, i.e., metals that increase blood glucose levels, which could be due to increased gluconeogenesis, damage to β-cells leading to reduced insulin production, or insulin resistance at target tissue resulting in a lack of glucose uptake. This review addresses the current evidence for the role of Cd, leading to insulin resistance from human, animal, and in vitro studies. Available data have shown that Cd may affect normal insulin function through multiple pathways. There is evidence that Cd exposure results in the perturbation of the enzymes and modulatory proteins involved in insulin signal transduction at the target tissue and mutations of the insulin receptor. Cd, through well-described mechanisms of oxidative stress, inflammation, and mitochondrial damage, may also alter insulin production in β-cells. More work is necessary to elucidate the mechanisms associated with Cd-mediated insulin resistance.

## 1. Introduction

Insulin-mediated glucose disposal widely varies in its sensitivity across populations [1], and depending on the level of compensatory hyperinsulinemia, resistance to insulin can or cannot be overcome. This insensitivity can lead to glucose intolerance, high-plasma triglyceride levels, low high-density lipoprotein cholesterol (HDL-C) concentrations, and hypertension [2]. If not overcome, it will lead to type 2 diabetes (T2D) development. Simultaneously, this collection of abnormalities has been linked with a significantly increased risk of cardiovascular diseases (CVD) [2]. The proposed association was formally established in the report of the adult treatment panel III of the National Cholesterol Educational Program. Formerly known as syndrome X, insulin resistance (IR) syndrome represents the insensitivity of the peripheral tissues (e.g., muscle, liver, adipose tissue) to the effects of insulin. IR is defined as a state wherein normal insulin concentrations evoke a less-than-normal biological response [3]. Although not a disease per se, it may be understood as a condition that increases the likelihood of developing a cluster of abnormalities, such as glucose intolerance, dyslipidemia, endothelial dysfunction, hemodynamic changes, increased testosterone secretion, and sleep-disordered breathing [4]. Additionally, it does not necessarily lead to, but instead increases the risk of clinical syndromes like CVD, essential hypertension, polycystic ovary syndrome, nonalcoholic fatty liver disease, and certain forms of cancers [2]. Metabolic syndrome (MS) is a collection of cardiometabolic risk factors. It is often characterized by IR that may provide a link between physical inactivity and MS development [5]. Most importantly, IR and impaired insulin secretion play a crucial role in the pathogenesis of T2D [6]. Although the potential causative connection has been shown for certain pollutants and IR development, the role of environmental chemicals in IR pathogenesis and the molecular mechanisms contributing to its development have not been fully elucidated yet [7].

Over fifty years ago, there was evidence suggesting that certain inorganic elements may alter glucose utilization in target tissues via sulfhydryl modification [8]. Since the 1970s, there has been a growing body of evidence that supports the involvement of these elements in various metabolic disorders associated with impaired β-cell function [9,10,11,12]. Several inorganic elements may influence the proper regulation of insulin/glucose homeostasis. They can be divided into two categories—hyperglycemic and hypoglycemic—based on their effects on insulin production or insulin action at target tissues [13]. The categorization of different elements is displayed in Table 1.

Our interest is focused on the actions of cadmium (Cd) and its ability to alter numerous cell and organ systems. This toxic metal is characterized by a high soil-to-plant transfer rate, which makes the dietary exposure to this metal inevitable and a matter of great public health concern [14,15]. Another important source of exposure of the general population to Cd is smoking, as shown by elevated Cd levels in the smokers’ blood [16]. Once inside the organism, Cd has a long biological half-life, with estimates reaching 45 years for humans [17]. Whole-blood and urinary Cd concentrations are widely accepted markers of Cd exposure and accumulation [14,18]. Long-term exposure to Cd has been associated with various conditions, including various renal syndromes, osteoporosis and osteomalacia, CVD, and different types of cancer [14,15,19,20,21,22,23,24,25]. Its endocrine-disrupting properties have also been shown, suggesting its possible effects on estrogenic activity [26,27,28,29], alterations in semen and the testis [30,31,32], and a role in thyroid disorders [33,34,35]. The mode of toxic Cd actions in the organism have been extensively investigated, but still not entirely elucidated, mainly because they may change with the dose and the detailed health status of the exposed subjects. Recent reviews by Đukić-Ćosić et al. [36] and Wallace et al. [37] have summarized the most critical mechanisms of Cd toxicity: changes in gene expression and DNA repair, interference with autophagy and apoptosis pathways, oxidative stress induction, interaction with bioelements, and epigenetic modifications. These mechanisms underlie the possible role of Cd as a metabolic disruptor. Its direct pancreatotoxic actions are buttressed by the Cd’s ability to accumulate in the pancreas, as shown in many human studies [22,38,39,40]. Similar results have been obtained in animal studies as well [41,42], with a dose-dependent accumulation pattern observed in rats [22]. In vitro studies have shown not only dose- but also a time-dependent accumulation of Cd in insulin-producing β-cells [43]. Furthermore, having in mind Cd’s deleterious effects on the kidneys [14] and the role of kidneys in glucose homeostasis, which is accomplished through the processes of gluconeogenesis, glucose filtration, glucose reabsorption, and glucose consumption [44], it could be presumed that Cd’s effects in the kidneys do contribute to IR development to a certain point. One of the first reports of Cd’s ability to promote the development of diabetes appeared nearly four decades ago, when Merali and Singhal reported that neonatal exposure to Cd resulted in IR and diabetes development in rats [45]. The present review aims to provide an overview of the potential role of Cd exposure in IR collected in human, animal, and cell studies, focusing mainly on those conducted in the last two decades. Furthermore, the review will also briefly discuss the existence of a threshold for this effect.

## 2. Insulin Resistance and Cadmium: Human Studies

Human studies investigating the link between Cd exposure and IR are limited and have yielded somewhat conflicting data. The first association between Cd content, impaired fasting glucose (IFG), and diabetes was suggested by Schwartz et al. [46], who analyzed the data of the third National Health and Nutrition Examination Survey (NHANES III). This large, cross-sectional study revealed a significant, dose-dependent association between Cd urinary levels and IFG/diabetes prevalence, regardless of the source of Cd exposure. Another study analyzing NHANES participants for the years 2005 through 2010 aged ≥40 years revealed a complex, non-linear association between higher Cd levels and prediabetes state. Since this association varied across smoking groups and age, the authors suggested a complex relationship between Cd exposure, age, smoking habits, and prediabetes odds. Nevertheless, since no differences in the Homeostatic Model Assessment for IR (HOMA-IR) were observed across the exposure quintiles, the authors marked changes in IR as an unlikely cause of Cd effects on glucose levels [47]. The relationship between Cd exposure and T2D occurrence was confirmed in the study comparing the levels of Cd in various biological samples (blood, urine, and scalp hair) of patients having T2D (age range 31–60) with the levels in control subjects. Significantly higher levels of Cd were observed in scalp hair samples from patients compared to control individuals, along with a similar trend in observed values obtained from blood and urine samples [48]. Studies that followed tried to establish possible mechanisms of Cd in disturbing glucose metabolism. Pizzino et al. [49] investigated glycemic control, oxidative stress markers, and urinary Cd levels from 111 males (aged 12–14 years) living in polluted areas of Sicily and control age-matched population of 60 males living 28–45 km from the contaminated site. The results revealed altered glycemic control in adolescents that was associated with higher Cd levels. Altered glycemic control was demonstrated by the robust correlation between Cd and the homeostatic model assessment of HOMA-IR, along with markers of disturbed oxidative status. The authors identified oxidative stress disturbance to play a role in Cd-induced IR [49]. Apart from oxidative stress induction, Cd’s ability to induce inflammation was also investigated. In a case-control, cross-sectional study, including 120 healthy controls and 105 systemic lupus erythematosus (SLE) patients, the relationship between various trace elements with SLE diagnosis, disease activity, and IR was assessed [50]. Serum levels of Cd were higher in patients with IR. Cd’s ability to impair insulin sensitivity was connected to the positive association between Cd and the C-reactive protein (CRP). Namely, CRP as an inflammatory marker was shown to have a role in the development of diabetes [51].

Conflicting results from multiple studies have complicated the interpretation of Cd-mediated hyperglycemia. Jacquet et al. provided a comprehensive review of these conflicting reports in a 2016 review, where they categorized the effects as having “associations”, “no association”, or “potentiation” in diabetes [52]. Examples of this data variability is reflected in a study of Scandinavian Caucasian women, aged 64, which showed conflicting data with the previously mentioned studies [53]. Two thousand five hundred and ninety-five women were screened with oral glucose tolerance tests to identify subjects with T2D, impaired glucose tolerance (IGT), and normal glucose tolerance (NGT), and samples were randomly chosen from each group. Cd concentration was measured in blood and urine samples, while the HOMA-IR calculations assessed the acute insulin response. A follow-up examination was also performed. Both cross-sectional and prospective studies showed no association between Cd exposure and increased risk of T2D, impaired insulin secretion, or insulin sensitivity [53]. The discrepancies in these results with previously published data were explained by differences in the occurrence of T2D risk factors in investigated groups, such as age, obesity, smoking, lifestyle, and ethnic predispositions. Furthermore, all women studied were aged 64+, which does not inform on the behavior of other populations. Interestingly, however, Wu et al. [54], in their PRISMA-compliant systematic review and meta-analysis based on 11 cohort/cross-sectional studies included in the meta-analysis, determined that high Cd exposure may not be a risk factor for diabetes development. Moreover, Anetor et al. [55] found significantly lower Cd blood levels from diabetic patients when compared to controls. This conflicted finding was partly attributed to the observed higher Zn levels in the same group of patients and the relatively small sample size (65 participants). Indeed, Cd belongs to the same group of elements as zinc, and the number of common biological targets of the two metals abound [56,57,58,59,60]. The role of zinc in insulin regulation has been extensively examined [61,62,63]. Zinc associates with insulin in exocytosis granules, and a significant amount of zinc is subsequently released into the extracellular space. Zinc can act as an autocrine mediator, affecting the activity of surrounding β-cells [61]. Defects in the transporters, such as SLC30A (ZNT) for the Zn provision for insulin secretion or SLC39A (ZIP) for replenishment, result in reduced intracellular zinc and a reduction in functional insulin release [62]. Changes in functional insulin release and alterations in zinc homeostasis may thus combine to contribute to glucose intolerance and IR [63].

Swaddiwudhipong and associates conducted a series of studies in Cd-exposed adults from Mae Sot District, Tak Province, in northwestern Thailand. This region was contaminated by the Cd-rich waste of Zn mines, and the population was Cd-exposed by the consumption of rice and other crops irrigated by downstream water. No association between urinary Cd levels and an increase in diabetes prevalence and risk were found [64,65]. The follow-up examination conducted on 436 persons who had urinary Cd levels >5 µg/g creatinine revealed a significant increase in the prevalence of diabetes compared to baseline [66].

The question of the role of kidneys in Cd-induced IR has been raised earlier. The kidney is responsible for up to 20% of all glucose production, and these figures are even higher in diabetic conditions [44]. Moreover, IR represents an early metabolic alteration in chronic kidney disease (CKD) patients, with the skeletal muscle representing the primary site of IR [67]. On the other hand, a recent study in a group of 395 subjects from low- and high-Cd exposure areas demonstrated that glomerular filtration rate could be linked to Cd exposure and tubular toxicity [68]. This linkage was shown to act in both dose and toxicity severity-dependent manners. The association of Cd with CKD was recently highlighted in a review by Satarug [14], which addressed the connection between Cd dietary intake and its effects on kidneys. However, animal studies have shown that the Cd effect on fasting blood glucose elevation is evident before signs of renal dysfunction are overt [69]. It is, nevertheless, highly plausible that Cd acts synergistically with chronic hyperglycemia seen in diabetic nephropathy. For example, research on 65 participants, consisting of 45 T2D and 20 healthy individuals, revealed the association between higher Cd levels in the poor glycemic control group [55]. Thus, Cd should certainly be considered as the agent of high importance in the progression of diabetes-related kidney disease, and the toxic effects of Cd in the kidney certainly further contributes to the role of Cd in IR.

Studies conducted in human subjects have shown conflicting data on the role of Cd in IR development. The obtained results depend on many factors, and the actual prevalence of diabetes in the study population seems to have an important impact on them. Although questionable due to ethical reasons, prospective studies investigating the Cd levels, especially low-level exposure, before the presentation of pathologies/toxicity is warranted to establish the causality bases for this association.

## 3. Insulin Resistance and Cadmium: Animal Studies

For the last five decades, studies in animals have suggested that both acute and chronic Cd exposure can affect glucose metabolism and synthesis regulation, and alter insulin secretion [70,71]. Intraperitoneal administration of a single dose of Cd-acetate to mice (2.0–6.0 mg/kg body weight (b.w.) and rats (0.84 mg/kg b.w.) was shown to cause a significant increase of blood glucose [70,72]. In addition, feeding animals chow containing increasing amounts of Cd (0–200 ppm CdCl_2_) for 30 days resulted in a significant dose-dependent elevation of blood glucose levels [73]. The ability of the pancreas to accumulate Cd has been demonstrated in multiple animal studies. Chronic oral administration of Cd for 60 days (100 mg/L) in rats resulted in the accumulation of the metal in the pancreas and a significant decrease in serum insulin levels, followed by a reduction in insulin gene expression [74]. Similar results were obtained after a single oral exposure to a high dose of Cd in rats, where the pancreas accumulated this toxic metal [22]. Studies directed at investigating the impact of environmentally relevant doses of Cd showed that Cd exhibited gender-specificity in glucose metabolism disruption, with females being more sensitive [75]. The same authors demonstrated that low-level maternal exposure to Cd influences glucose homeostasis in offspring and increases the risk of offspring developing T2D later in life [76].

Although different animal experiments indicate impaired glucose metabolism, insulin secretion, and tissue resistance to insulin, the exact mechanisms of these effects of Cd are still hardly known. Under physiological conditions, the maintenance of glucose homeostasis depends on a coordinated process of balancing circulating glucose levels and the release of insulin by the pancreatic β-cells. In the post-absorptive state, 75% of glucose uptake occurs in the insulin-independent tissues, mainly in the liver and brain tissue. In comparison, the remaining glucose uptake (25%) occurs in insulin-dependent tissues, as well as muscle and adipose tissue [77]. To some extent, β-cells can compensate hyperglycemic states by elevating the secretion of insulin at the expense of the likelihood of IR development in multiple peripheral tissues [78].

Based on the studies in animal models of Cd exposure, Cd can produce a direct effect on the pancreas and affect glucose transport in insulin-independent and insulin-dependent tissues. Numerous animal studies have demonstrated that Cd exposure influences glucose metabolism by directly affecting pancreas morphology and β-cell function, resulting in cellular damage. Furthermore, oxidative damage, as a known phenomenon important in diabetes development, may occur upon Cd accumulation [52].

A hyperglycemic state in animals involves the increased activity of the glycogenolysis pathway and stimulation of enzymes associated with the gluconeogenesis pathway [73]. Apart from the Cd effects on gluconeogenesis, Cd can influence insulin via different patterns. Lei et al. [74] demonstrated that subcutaneously administered cadmium (0.5, 1.0, and 2.0 mg/kg b.w.) decreased insulin gene expression in exposed rats. This investigation suggests that Cd can influence the biosynthesis of insulin but has no effects on its release. Additionally, the same study revealed pancreatic dysfunction occurring earlier than kidney dysfunction following Cd administration.

Apart from the direct Cd effect on pancreatic β-cells, exposure to this metal affects glucose transport in other tissues, all potential sites of Cd toxicity. In these tissues, insulin exhibits different effects: in skeletal muscle, it promotes glucose utilization and storage by increasing glucose transport and net glycogen synthesis; in the liver, it activates glycogen synthesis, increases lipogenic gene expression, and decreases gluconeogenic gene expression, whereas, in white adipocyte tissue (WAT), it suppresses lipolysis and increases glucose transport and lipogenesis [79,80]. Glucose uptake in different animal cell types is mediated by a family of intrinsic membrane proteins (products of the *GLUT*/*SCL2A* genes) that facilitate glucose transport through membranes. GLUT4, the insulin-responsive glucose transporter, is selectively produced in muscle and adipose cells, while GLUT2 occurs in hepatic cells [81,82].

Generally, in skeletal muscle, Cd-induced IR suppresses glucose utilization and storage by decreasing glucose transport and net glycogen synthesis [79,80]. One-month administration of CdCl_2_ (50 mg/L) with drinking water in male rats resulted in a significant decrease in plasma insulin-like growth factor 1 (IGF-I) and insulin-like growth factor binding protein-3 (IGFBP-3) levels [83], factors known to be altered in IR. Insulin resistance in the muscle of rats chronically exposed to Cd has been associated with the reduction of glycogen synthesis. Studies with rodents exposed to Cd have shown decreased GLUT4 expression, which could partly explain the reduction in glycogen synthesis [79].

In the liver, insulin activates glycogen synthesis, increases lipogenic gene expression, and decreases gluconeogenic gene expression. Cadmium-induced IR in the liver leads to a significant increase in hepatic GLUT2, carbohydrate regulatory element-binding protein, glucokinase, and pyruvate kinase mRNA [84]. Zhang et al. [85] have demonstrated that animals exposed to Cd developed IR as the result of the activation of lipogenic proteins, leading to a significant increase in serum glucose and free fatty acids.

Finally, in adipose tissue, especially white adipocytes, insulin suppresses lipolysis and increases glucose transport and lipogenesis [80]. Thus, the adipose IR is the inability of insulin to activate adipose glucose transport, promote lipid uptake, and suppress lipolysis [86]. Several mechanisms were suggested to contribute to the adverse Cd effects on adipose tissue pathophysiology and subsequently increased IR. Han et al. [79] demonstrated that subacute administration of Cd (subcutaneously. 2 mg/kg daily for four days) produces impaired glucose tolerance (IGT) in rats, which was associated with a dose-dependent reduction in GLUT4 protein and GLUT4 mRNA levels in adipocytes. Furthermore, the IR state in adipose tissue favors lipolytic pathways, resulting in an elevation of free fatty acids (FFA), which additionally contributes to impaired insulin secretion when released in the plasma.

## 4. Insulin Resistance and Cadmium: Cellular Studies

### 4.1. Non-Pancreatic Cells

Many in vitro studies have used non-pancreatic cell lines, such as adipocytes or ovarian/granulosa cells, that are typically involved with insulin action or glucose utilization [87]. In adipocytes directly obtained from Wistar rats, exposure to 5 µM Cd increased the cellular metabolism of glucose, similar to zinc, but did not increase glucose uptake, which is the opposite of zinc’s action [88]. The primary glucose transporter stimulated by insulin is GLUT4, and an early study suggests that Cd exposure decreases the activity of the GLUT1 transporter [89]. Exposure to Cd also reduces critical cellular mediators involved in the differentiation and normal function of adipocytes. Decreased leptin, adiponectin, and resistin alter cellular ability to normally process lipids, potentially leading to IR [90,91]. Proteins that interact with the motif cytosine–cytosine–adenosine–adenosine–thymidine (CCAAT) are referred to as “CCAAT-enhancer-binding proteins” and are a target of Cd action in adipocytes. There are six CCAAT-enhancer-binding proteins involved in normal adipogenesis, including β and δ, which are activated in early adipocyte differentiation, and α, which is upregulated in the later stages of adipogenesis. Early in adipogenesis, β and δ stimulate peroxisome proliferator–activator receptor-γ (PPARγ). Exposure to Cd has been shown to inhibit the production of both CCAAT-binding enhancer proteins and PPARγ, leading to IR and an increase in adipogenesis [91,92]. In adipocytes, exposure to Cd alters the cellular functions involved with lipid metabolism and IR development, leading to obesity or diabetes.

### 4.2. Pancreatic Cells

Of the various elements, arsenic is possibly the most studied with regard to its effects on pancreatic function and the subsequent development of diabetes. Directly comparing arsenic, manganese, and Cd actions, the reduction in glucose-stimulated insulin section after arsenic and manganese exposure appeared to be due to mitochondrial dysfunction. In contrast, the inhibitory effects observed following Cd exposure were due to a mitochondria-independent mechanism [9]. There is evidence that mitochondria can transport Cd via the calcium uniporter, resulting in interference of the K+/H+ exchanger [93]. Nearly forty years ago, a transport-specific deficiency was identified due to changes in Cd sensitivity encoded by the *SLC39A8* gene [94]. The transporter encoded by this gene is highly conserved across species and has been shown to encode a specific element cation transporter referred to as *ZIP8*. Examination of β-cell function after arsenic, manganese, and Cd exposure revealed a distinct pattern of miRNA expression changes unique to each element, suggesting that biochemical differences result in distinct responses. Exposure to inorganic arsenic gave rise to a significant 76% increase in miR-146a, while there was a 60% decrease in miR-195 expression following Cd exposure [95]. Since β-cells are excitable cells and involve cell depolarization, leading to insulin release, several studies have examined the effects of metal exposure on voltage-gated calcium channel function. In an early study, ex vivo, isolated, perfused rat pancreas demonstrated that the addition of Cd to the perfusion buffer reduced insulin release, possibly due to the blockade of calcium uptake, thus preventing β-cell depolarization [96]. Characterization of the calcium channels suggests that the L-type (long-lasting) calcium channel is the predominant channel on β-cells, and that Cd acts by preventing calcium uptake via L-type channels [97]. Voltage-dependent calcium uptake is dependent on β-cell depolarization via potassium channel activity. Cd-mediated effects on potassium channels appear negligible and are mediated by voltage-dependent calcium channel inhibition [98]. In addition to blocking calcium uptake, Cd itself can be transported into β-cells and accumulate within the cells. Interestingly, at relatively low concentrations (5 µM), in the absence of calcium applied for one hour, the non-stimulated insulin release from islets of obese–hyperglycemic mice increased, but not the glucose-stimulated one [99]. Changes in insulin release in the presence of 5 µM Cd appears to be independent of calcium involvement. Lower concentrations of Cd do not increase intracellular calcium, nor inositol 1,4,5-triphosphate (IP_3_) [100]. When 1 h-applied Cd concentrations exceed approximately 160 µM, insulin release is inhibited [99]. One conclusion from earlier studies is that Cd acts like a “silent killer”, being taken up into β-cells and accumulating with time. During the early stages, β-cells would function normally until the Cd detoxification systems are overcome. Cd content from normal, “non-diabetic” human β-cells is approximately 29 nmol/g protein, with significant variability between individuals; that is not high enough to impair normal pancreatic function, as indicated by the lack of diabetic symptomology [43]. Determining intracellular Cd concentration and correlating intracellular values to extracellular concentrations have been challenging. Mathematical modeling data obtained from intestinal cell lines suggests that an external concentration of 10 μM would lead to an intracellular Cd concentration of 5000 amol/cell [101]. Cd continuously accumulates over time, at concentrations that may not significantly alter cell viability or gene expression [43]. Changes in β-cell function at sub-lethal concentrations seem to involve mitochondrial adaptation. As Cd accumulates in β-cells to several hundred-fold over baseline concentrations, mitochondria begin to appear fragmented, with the fusion–fission state shifting towards fission [102]. Studies using the INS-1 human pancreatic β-cell line utilized Cd concentrations 10-fold below the threshold necessary for cell death. Concentrations of Cd that are subtoxic produced no effects on mitochondrial function that were assessed by the energy change and the synthesis of adenosine triphosphate (ATP). Yet there were no morphological changes, suggesting a mitochondrial adaptive response to low-level Cd. The authors concluded that if cellular Cd influx continues, impairment of this organelle may contribute to cellular dysfunction and decreased viability of β-cells [102].

Mitochondrial respiration (oxygen consumption) and energy state (adenosine triphosphate production) appear unchanged during this process, until the cells commit to the death pathways [93]. Disruptions of mitochondrial morphology and energy state are linked to the onset of apoptosis. The intracellular mechanisms associated with Cd-mediated apoptosis have not been completely elucidated. The intracellular apoptotic mechanisms usually co-exist with necrosis, with a proportion of each depending on the cadmium dose and other conditions [102]. Exposure to Cd has been shown to elevate oxidative stress in pancreatic cells [103]. Increases in oxidative stress are linked to increased levels of malondialdehyde, free cytochrome c, p53, extracellular-regulated kinases 1/2, p38-mitogen-activated kinase, and *c-jun* N-terminal kinase (JNK), but decreases in mitochondrial membrane potential and Bcl-2 have also been observed [104]. Of the changes observed following Cd exposure, the increase in JNK activity by increased oxidative stress has been postulated to be one trigger for apoptosis. Not only is the phosphorylation of JNK upregulated after just one hour of exposure to 10 µM Cd, the expression of CCAAT-enhancer binding protein homologous protein, CHOP, is significantly upregulated [105]. Mitogen-activated kinases are vital for the normal function of the cell through proliferation, differentiation, and apoptosis. CHOP has been linked as an apoptotic response to oxidative stress in the endoplasmic reticulum. Additionally, Cd-mediated effects have been demonstrated directly via activation of the extracellular, signal-regulated kinases (ERK1/2) [106,107]. In general, ERKs activate numerous downstream pathways and are involved in Cd-induced carcinogenesis [108,109]. Together, reported effects of Cd on β-cells provide different pathways for Cd-mediated responses, ultimately leading to apoptosis, cell death, and a lack of β-cell responsiveness to elevated glucose, leading to stimulated insulin release.

## 5. Insulin Resistance and Cadmium: Is There a Threshold?

Considering Cd-mediated IR, the critical question is as follows: is there is a threshold for this effect? As recently reviewed [110], uncertainties in the mechanisms of low-level metal toxicity for humans and the demonstration of the existence of a safe threshold remains a rather challenging issue in toxicology. The comparable issue of whether endocrine disruption (ED) occurs at a threshold value has been holding for years, and the scientific community still expresses controversial views. The Endocrine Disrupters Expert Advisory Group (ED EAG) of the European Commission published a report in 2013 [111] summarizing experts’ opinions regarding the existence of ED thresholds of adversity and the possibility of estimating such thresholds from existing experimental data. Most experts expressed the view that a threshold is likely to exist, but it might be exceptionally low. The basis of this argument is that one molecule, bound to only one receptor, would not be enough to activate the cascade of events needed to lead to apical adversity. However, in the case of fetal development, a threshold might not exist, due to the immaturity of the endocrine system [112]. Furthermore, with regard to the possibility of non-monotonic dose-response curves (NMDRCs), the available assays and methodologies most probably are not adequate for estimating a threshold with sufficient accuracy and sensitivity [113]. Other experts have expressed the view that a threshold does not exist, as endogenous hormones are already present in the body, and only one molecule of a xenobiotic might be enough to overwhelm the homeostatic system [114,115]. Based on these arguments, the EDs’ risk assessment in most cases in the European Union is based on a hazard-based (no-threshold) approach, although a different, case-by-case approach for the Registration, Evaluation, Authorisation and Restriction of Chemicals (REACH) regulation has been proposed [116]. Other countries, such as the United States, Canada, and Australia, have adopted a risk-based threshold approach to overcome the difficulties discussed previously [117,118].

The estimation of a threshold under which Cd does not precipitate IR (further than the theoretical discussion of existence or not of such a threshold) would necessitate the existence of appropriate validated assays. For the moment, the globally existing recognized and accepted testing approaches cover modalities related only to the estrogen, androgen, thyroid, and steroidogenesis (EATS) pathways, as described in the OECD’s Conceptual Framework (CF) for Testing and Assessment of Endocrine Disrupters [119]. Assays designed explicitly for the IR are not available in clinical practice or the field of toxicology. However, we know that IR, at least as a syndrome, generates obesity, hypertension, high glucose, triglycerides, and an increased LDL/HDL ratio in the blood. These endpoints should be determined using validated assays, like the repeated dose 90-day study (OECD Test Guidelines (TG) 408) and chronic toxicity and carcinogenicity studies (OECD TGs 451, 452, and 453).

In a regulatory context, for setting an experimental threshold for Cd causing IR, the “no observed adverse effect level” (NOAEL) or, better, a benchmark dose (BMD) should be determined. To set a BMD, it should be defined (a) if adversity is considered only as the apical effect(s) or even earlier signs of effect, and (b) the exact values for the various endpoints and conditions that compose the IR as a syndrome. In relation to the apical adversity, as mentioned above, a practical approach could be to consider as such the combination of obesity, hypertension, high glucose, triglycerides, and increased LDL/HDL ratio in blood, as in clinical practice. In vivo experiments would be necessary, and consequently specific values that consider adverse effects should be set for animal studies (considering that an appropriate model exists). The available studies with rats and mice mentioned in the review of Tinkov et al. [84] have various shortcomings (such as very low duration, single dose used, use of engineered animals, and lack of adequate endpoints) to support the establishment of a NOAEL.

Additionally, the Cd mode of action or the related adverse outcome pathway should be elucidated, and adequate in vitro mechanistic assays should be developed and performed to prove causality. The exact cause(s) of IR is not yet known. Still, there are data supporting perturbations of the enzymes and modulatory proteins involved in insulin signal transduction. Oxidative stress, inflammation, insulin receptor mutations, endoplasmic reticulum stress, and mitochondrial dysfunction affecting the insulin-dependent cells of skeletal muscle and adipocytes have been put forward [120,121]. The study of which of the above mechanisms are the most sensitive to Cd and the possible existence of NMDRC through appropriate methodology are essential steps for substantiating the role of Cd in IR, and eventually proposing a scientifically robust and reliable threshold if any can be validated.

## 6. Conclusions and Remarks for Future

Even though evidence abounds suggesting the damaging role of Cd on glucose homeostasis, the debate continues over the importance of Cd toxicity in the increasing occurrence of diabetes. Conversations are ongoing about the preventive or curative measures that should be taken within the susceptible populations. Cd’s biological harm has been documented at different levels, from cells to human populations. However, the ample literature on cadmium toxicity (more than 15,000 articles are referenced in PubMed) covers such a variety of experimental conditions and formats that it is difficult to draw convincing strong conclusions from the available data.

**Figure 1:**
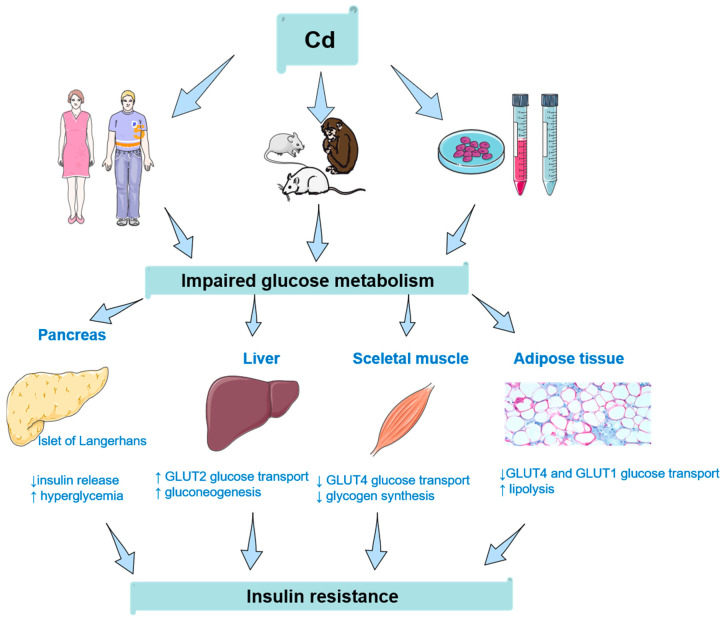
Cadmium’s (Cd’s) role in insulin resistance and glucose tolerance/impaired glucose metabolism in various organs: summary of the results obtained from human, animal, and cell studies. Insulin resistance appears first, followed by impairment of glucose metabolism. Cd’s effects in the kidneys do contribute to IR development to a certain point. In the pancreas, Cd accumulates in insulin-producing β-cells, leading to a decrease of insulin release and an increase in blood glucose in blood. Besides the direct effect of Cd on the pancreas, this toxic metal affects glucose transport in insulin-independent (liver) and insulin-dependent tissues (skeletal muscle and adipose tissue).

The main topic of the present review, namely IR, is no exception. Cd doses, origins of cells, and detailed information on the exposed populations are a few of the numerous variables that change and impair comparison between studies from different groups. At the time of writing this, we are still unable to propose a range of possible toxicity mechanisms, without knowing whether any level of exposure to environmental Cd can be accepted for human populations. Figure 1 briefly summarizes the role of Cd in impaired glucose metabolism in various organs.

Forthcoming studies should thus focus on the application of environmentally realistic doses in experimental studies since chronic low-level exposure of humans to Cd is seemingly inescapable. For its contribution to IR, parallel data would have to be obtained with optimized cellular models for each insulin target tissue. Meanwhile, cells and tissues involved in insulin turnover, secretion, and withdrawal would have to be similarly studied. The data at hand should now be enough to reach an international consensus on which concentration, (bio)chemical form, and duration of exposure should be implemented. In this process, negative results, i.e., a lack of observed effects as long as state-of-the-art methods are used, are as useful as positive ones. Since prospective population studies are precluded for obvious ethical reasons, and in the face of the skyrocketing diabetes development worldwide, it may be hoped that the above reductionist approach can define the most sensitive and useful markers of minute exposure for humans. This way, reliable monitoring of Cd-associated IR may be reached, and the degree of usefulness of preventive or corrective measures can be knowledgeably addressed.

## Figures and Tables

**Table 1:** Categorization of different elements as hyper- or hypoglycemic [13].

Hyperglycemic	Hypoglycemic
Arsenic	Zinc
Mercury	Vanadium
Iron	Chromium
Lead	Magnesium
Nickel	
Cadmium	
